# Design and synthesis of TiO_2_/C nanosheets with a directional cascade carrier transfer†

**DOI:** 10.1039/d2sc01872a

**Published:** 2022-05-10

**Authors:** Si-Ming Wu, Yi-Tian Wang, Shi-Tian Xiao, Yan-Xiang Zhang, Ge Tian, Jiang-Bo Chen, Xiao-Fang Zhao, Christoph Janiak, Menny Shalom, Detlef W. Bahnemann, Li-Ying Wang, Xiao-Yu Yang

**Affiliations:** State Key Laboratory of Advanced Technology for Materials Synthesis, Processing & Shenzhen Research Institute & Joint Laboratory for Marine Advanced Materials in Pilot National Laboratory for Marine Science and Technology (Qingdao), Wuhan University of Technology Wuhan 430070 China xyyang@whut.edu.cn; School of Chemical Engineering and Technology, Sun Yat-sen University (Zhuhai) Zhuhai 519000 China; School of Engineering and Applied Sciences, Harvard University Cambridge MA 02138 USA xyyang@seas.harvard.edu; Institut für Anorganische Chemie und Strukturchemie, Heinrich-Heine-Universität Düsseldorf Düsseldorf Germany; Department of Chemistry and Ilse Katz Institute for Nanoscale Science and Technology, Ben-Gurion University of the Negev Beer-Sheva 8410501 Israel; Institut für Technische Chemie, Leibniz Universität Hannover Callinstrasse 3 Hannover D-30167 Germany; Laboratory “Photoactive Nanocomposite Materials” (Director), Saint-Petersburg State University Ulyanovskaya str. 1, Peterhof Saint-Petersburg 198504 Russia; State Key Laboratory of Magnetic Resonance and Atomic and Molecular Physics, National Center for Magnetic Resonance in Wuhan, Wuhan Institute of Physics and Mathematics, Innovation Academy for Precision Measurement Science and Technology, Chinese Academy of Sciences Wuhan 430071 China

## Abstract

Directed transfer of carriers, akin to excited charges in photosynthesis, in semiconductors by structural design is challenging. Here, TiO_2_ nanosheets with interlayered sp^2^ carbon and titanium vacancies are obtained by low-temperature controlled oxidation calcination. The directed transfer of carriers from the excited position to Ti-vacancies to interlayered carbon is investigated and proven to greatly increase the charge transport efficiency. The TiO_2_/C obtained demonstrates excellent photocatalytic and photoelectrochemical activity and significant lithium/sodium ion storage performance. Further theoretical calculations reveal that the directional excited position/Ti-vacancies/interlayered carbon facilitate the spatial inside-out cascade electron transfer, resulting in high charge transfer kinetics.

## Abstract 2

Directional charge transfer in TiO_2_ nanosheets is achieved by design of TiO_2_ lattice-Ti vacancy-interlayered sp^2^ carbon at the interface.

## Graphical abstract

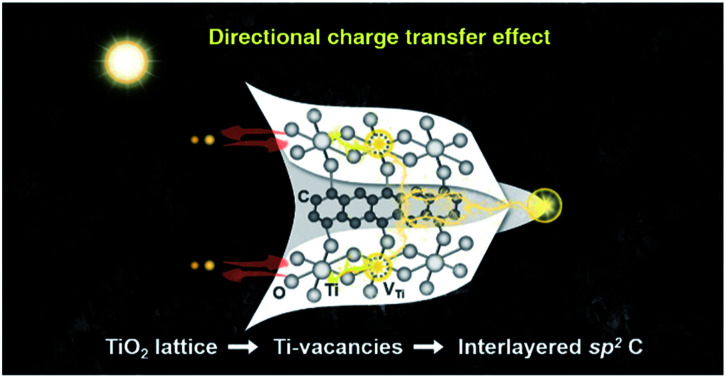

## 1. Introduction

Semiconductors are able to generate excited carriers that diffuse to the surface for photo/electro chemical reactions. The fast transfer and efficient utilization of carriers are critical to high performance in the desired application.^1–4^ Generally, the electron transfer at the single nanocrystal or interface of nanocrystals is random, often resulting in the electron dissipation and/or recombination before the electrons reach the surface of semiconductors for the redox reaction.^5,6^ Therefore, directed transfer of carriers, akin to excited charges in photosynthesis, enables a minimum of energy-loss and a maximum of energy-utilization. However, realizing directional charge transfer in semiconductors by structural design is challenging.

Titanium oxide (TiO_2_) has gained great attention as one of the most promising semiconductors for various photo and electrochemical applications.^7,8^ Carbon has been widely used to composite TiO_2_ as an electron acceptor for enhancement of the charge transfer.^9–11^ Carbonization in an inert atmosphere is a common way to fabricate the composition and doping structure.^12,13^ However, it will inevitably also form undesired carbon species such as low conductive sp^3^ carbon on the surface. Nanocarbon with a rich sp^2^ carbon structure, such as graphene sheets, enhances the conductivity and enables efficient charge transfer conductive performance in TiO_2_-based composites.^14,15^ However, the directed transfer of electrons only occurs in a limited range of the interfacial junction between TiO_2_ and nanocarbon in most cases.Recently, we have designed a spatially inside-out heterojunction with directed transfer of carriers, in which CQDs act as a bridge for interfacial charge transfer and graphene as a net for charge collection.^11^ It has to be pointed out that this ternary structure requires very careful adjustment and pre-formed nanocarbon with the sp^2^ structure. At high calcination temperature (over 1000 °C),^16^ the hydrocarbon precursor starts to transform into the graphitic sp^2^ structure, but at this temperature the size of the semiconductor particles, their crystal phases and morphologies would be greatly changed and even fused. Therefore, the common calcination method in an inert atmosphere is not suitable for the preparation of rich sp^2^ carbon in TiO_2_*via* precursor carbonization. Note that the calcination in air (so-called oxidative calcination) could easily lead to the removal of the surface carbon species of nanocomposites by oxidation at mild temperature (300–600 °C),^17^ but not to the removal of the carbon species from the crystal framework. Therefore, one theoretical possibility arises, namely that the oxidative calcination not only transforms titanium compounds into TiO_2_ and remove the unsatisfied surface carbon, but also forms inner rich-sp^2^ carbon confined by TiO_2_. The nanosheet structure recently developed is very elegant, which enables an abundance of vacancies and coordinatively unsaturated sites, shortens the mass diffusion length and greatly improves charge transfer performance.^18–20^ The rich-sp^2^ carbon structure in TiO_2_ nanosheets would therefore be a very effective design for directional charge transfer.

In this study, lamellar titanium glycerolate is chosen as the precursor for the synthesis of anatase TiO_2_ nanosheets with interlayered rich-sp^2^ carbon using a well-defined oxidization calcination process. The oxygen-rich environment contributes to the formation of titanium vacancies (Ti-vacancies). The directed transfer of carriers from the excited position to Ti-vacancies to interlayered carbon is investigated and proven to greatly increase the charge transport efficiency and electrical conductivity. Consequently, TiO_2_/C demonstrates significant photocatalytic and photoelectrochemical activity and excellent lithium/sodium ion storage.

## 2. Results and discussion

TGA-DTA of titanium glycerolate (denoted as Ti-G) (Fig. S1, ESI†) indicates the removal of surface carbon in the range of 300–339 °C and the phase transformation from anatase to the rutile structure at 435 °C. Therefore, we choose 350 °C for the synthesis of the metastable anatase phase TiO_2_/C composite without surface carbon, while the interlayered carbon species are still coordinated to TiO_2_ (detailed description in the ESI†). SEM images (Fig. S2a, ESI†) show that Ti-G is a flower-like particle with a uniform size of around 3 μm, and calcination leads to aggregation of nanocrystals in the branches (Fig. S2, ESI†). XRD patterns (Fig. S3, ESI†) and Raman spectra (Fig. S4, ESI†) indicate that calcination at 350 °C in air results in the phase transformation from titanium glycerolate to anatase TiO_2_. And calcination at 550 °C could lead to the formation of the rutile phase. N_2_ adsorption–desorption measurements (Fig. S5, ESI†) of the TiO_2_ sample calcined at 350 °C in air disclose a type II isotherm (indicative of non- or macroporous adsorbents) with an H3 hysteresis loop (due to non-rigid aggregates of plate-like particles).^21^ It also shows mesopores of around 3 nm and a large fraction of mesopores larger than 10 nm by the packing of the TiO_2_ nanocrystals, with a tube structure of 25 nm. Notably, the sample calcined at 350 °C in Ar has only 24 m^2^ g^−1^ of BET surface area with no obvious porous structure (Fig. S5 and Table S1, ESI†), because it has surface carbon that could block the pores.

**Fig. 1:**
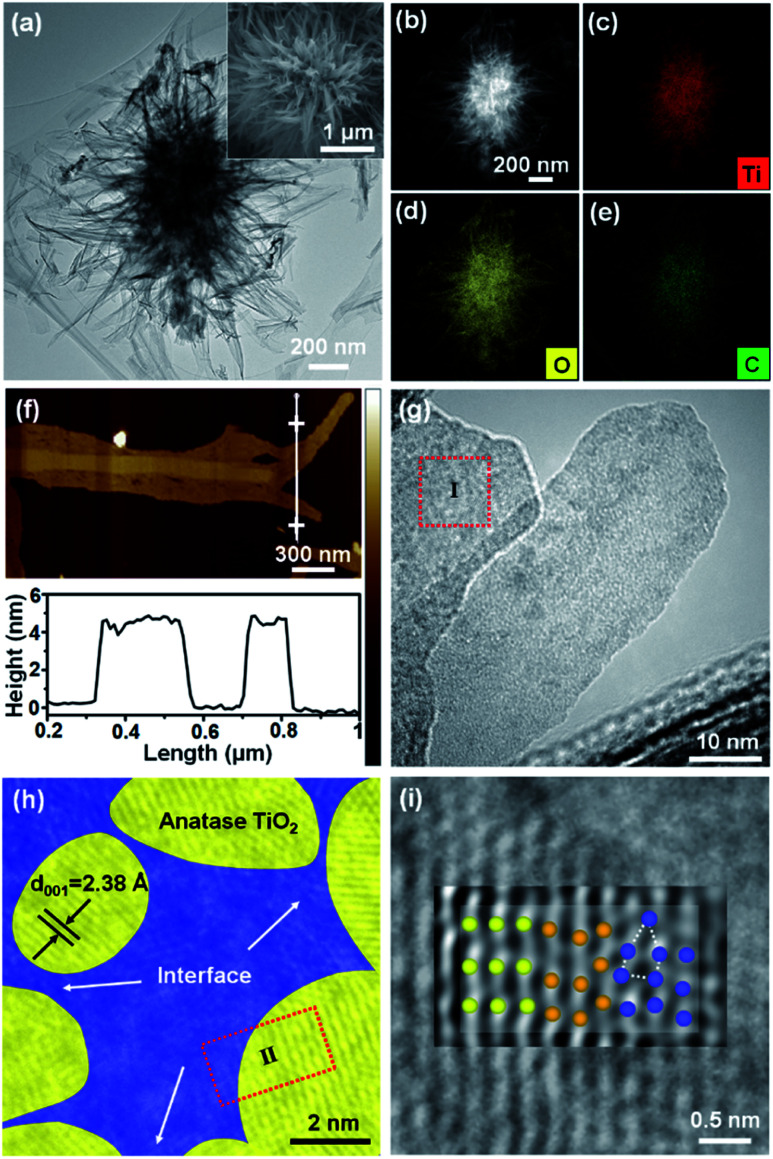
(a) TEM image, inset: SEM image, (b) STEM image and the corresponding EDX mapping image of (c) Ti, (d) O, and (e) C elements, (f) AFM topography image and the corresponding height information of TiO_2_/C_Inter_ (g) TEM image, (h) magnified TEM image of region I and (i) original image and inverse FFT image of region II, and the corresponding atomic models of the highly crystalline phase (ordered lattice, light yellow, left), nanofusion phase (dark yellow, middle), and semi-crystalline/amorphous phase (disordered defects, blue, right) of TiO_2_/C_Inter_. Fig. parts (h) and (i) are given in Fig. S7, ESI† as originals without the interpreting color overlay.

As shown in Fig. 1a and the inset, the flower-like structure of TiO_2_/C_Inter_ is assembled by very thin nanosheets. The STEM image and the corresponding EDX spectral mapping images (Fig. 1b–e) indicate the uniform distribution of Ti, C and O elements. The AFM tomography studies (Fig. 1f) show that the branches of the particle are nanosheets with a thickness of around 4–5 nm, which is in accordance with the magnified TEM image (Fig. S6, ESI†). The HRTEM image (Fig. 1h, S7a and b, ESI†) shows the lattice fringes with an interplanar spacing of 0.35 nm, which agrees with the (101) planes of anatase TiO_2_. The anatase TiO_2_ nanocrystals (Fig. 1h, yellow areas) are surrounded by an amorphous phase (Fig. 1h, blue areas). In the HRTEM image and the inverse FFT image (Fig. 1i and S7b–d, ESI†), the ordered lattice fringes (light yellow, left), the lattice distortions (dark yellow, middle) and the disordered amorphous phase (blue, right, probably involving amorphous TiO_2_ and carbon) are clearly recognized. This kind of crystalline/semi-crystalline/amorphous interface is coherent at the atomic scale and thus becomes a platform for the inner carbon and defect generation and minimizes interface loss of energy.

**Fig. 2:**
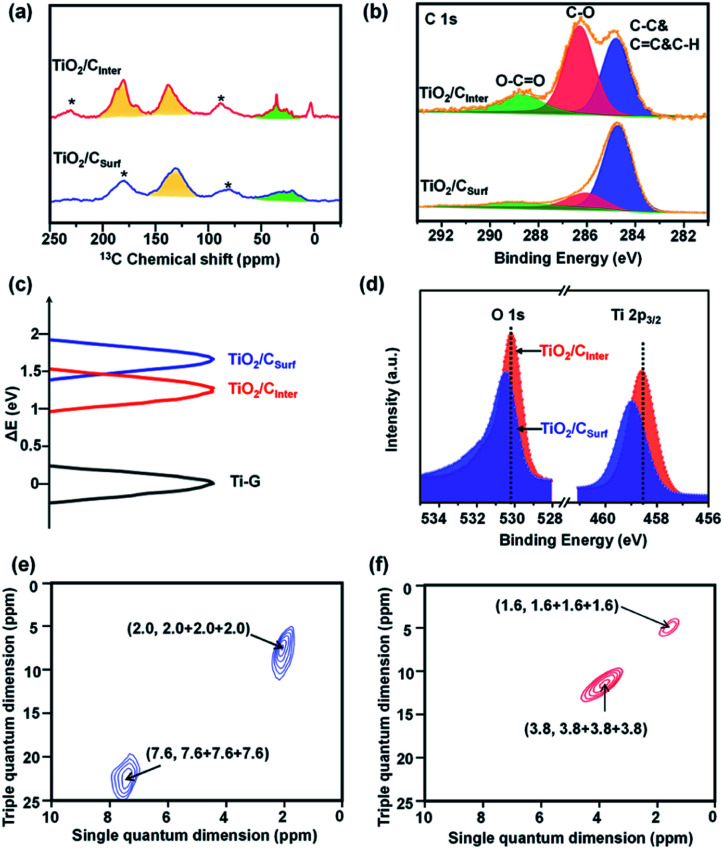
(a) ^13^C NMR spectra of TiO_2_/C_Inter_ and TiO_2_/C_Surf_, * indicates the signal of the rotor cap, (b) XPS C 1s spectra of TiO_2_/C_Inter_ and TiO_2_/C_Surf_, (c) Ti 2p_3/2_ chemical shift among Ti-G, TiO_2_/C_Inter_ and TiO_2_/C_Surf_. (d) Ti 2p_3/2_ and O 1s XPS spectra of TiO_2_/C_Inter_ and TiO_2_/C_Surf_, and (e and f) ^1^H TQ-SQ MAS NMR spectra of (e) TiO_2_/C_Inter_, and (f) TiO_2_/C_Surf_.

^13^C cross-polarization/magic-angle spinning NMR is an effective method to investigate carbon species comprehensively and quantitatively.^22–24^ For comparison, TiO_2_ mainly with surface carbon (named TiO_2_/C_Surf_, by calcination from Ti-G at 350 °C in an Ar atmosphere) is also characterized. Ti-G shows three well resolved peaks between 69 and 90 ppm (Fig. S8, ESI†), indicating the successful coordination of glycerol.^25,26^ The peaks near 138 ppm in TiO_2_/C_Inter_ (Fig. 2a and S9, ESI†) and 135 ppm in TiO_2_/C_Surf_ are assigned to sp^2^ hybridized C atoms.^24,27^ The peaks around 30–50 ppm are assigned to sp^3^ C atoms.^27^ Besides, TiO_2_/C_Inter_ shows an additional peak at around 180 ppm, which can be ascribed to C=O with sp^2^ hybridized carbon. Very interestingly, the ratio of sp^2^ and sp^3^ in TiO_2_/C_Inter_ is about 2.6-fold that of the TiO_2_/C_Surf_ (Fig. S9 and Table S2, detailed description in the ESI†), suggesting a high level of sp^2^ carbon in TiO_2_/C_Inter_. Raman spectra (Fig. S10, ESI†) show only graphite carbon in TiO_2_/C_Inter_ and the weak peak indicates the low content and inner structure of carbon species.^28^ The high-resolution C 1s spectra (Fig. 2b) could be deconvoluted as the C–C bond (around 284.8 eV), C–O–(Ti) bond (around 286.3 eV) and O–C=O (around 288.7 eV).^29,30^ There is an obvious difference of the peak area ratio of the C–C bond and C–O–(Ti) bond between TiO_2_/C_Inter_ (0.84) and TiO_2_/C_Surf_ (4.52) (Fig. 2b). Further evidence is provided by the surface carbon content and C/Ti ratio calculated from Ti 2p and C 1s XPS data (Fig. S11 and Table S3, detailed description in the ESI†). TiO_2_/C_Inter_ shows a low surface carbon content (15.0 at%) near to the sample calcined at 550 °C (15.5 at%), while TiO_2_/C_Surf_ shows a relatively high carbon content (44.7 at%) similar to the original carbon content in Ti-G (47.0 at%). These observations suggest that the surface carbon content can be reduced by oxidization calcination but the inner carbon species in TiO_2_/C_Inter_ would remain. This can also be observed in the FT-IR spectra (Fig. S12, detailed description in the ESI†). After low temperature calcination, TiO_2_/C_Inter_ exhibits a grey color with enhanced visible-light absorption and a narrower bandgap (Fig. S13, ESI†). The more positive Ti 2p chemical shifts of TiO_2_/C_Inter_ and TiO_2_/C_Surf_ indicate the increase of the electron–electron repulsion around the Ti atoms by strong interactions of the Ti–O–C bonds (Fig. 2c and d). The O 1s core-level XPS spectrum of TiO_2_/C_Inter_ (Fig. S11c, ESI†) displays the major peaks caused by the O^2−^ ions in the O–Ti–O lattice (around 530.0 eV).^31,32^ The O^−^ species (around 532.0 eV) are considered to correlate with the Ti-vacancies to compensate for the Ti^4+^ deficiencies.^33–35^ The formation of Ti-vacancies can be deduced from the surface O/Ti ratio of the samples (Fig. S11e and Table S3, detailed description in the ESI†). The EPR spectrum of TiO_2_/C_Inter_ (Fig. S14, ESI†) shows a strong signal at *g* = 1.998, which could be assigned to Ti-vacancies.^36,37^

The two-dimensional (2D) ^1^H TQ-SQ MAS NMR method is an effective way to identify the local structure and interactions in TiO_2_ by the investigation of titanol (Ti–OH) sites.^38,39^ The dipolar interactions of ^1^H can directly probe the spatial proximities of Ti–OH groups such as surface Ti–OH, Ti–OH from broken Ti–O–Ti bonds and Ti–OH nests from Ti vacancies. More importantly, the interlayered carbon could also affect the chemical state of the Ti–OH groups. The signal of surface Ti–OH groups in mutual spatial proximity is observed in both TiO_2_/C_Surf_ (2.0, 2.0 + 2.0 + 2.0) (Fig. 2e) and TiO_2_/C_Inter_ (1.6, 1.6 + 1.6 + 1.6) (Fig. 2f). Besides, TiO_2_/C_Surf_ shows a signal at (7.6, 7.6 + 7.6 + 7.6), which indicates that the Ti–OH species from broken Ti–O–Ti bonds are spatially close. Meanwhile, TiO_2_/C_Inter_ shows a signal at (3.8, 3.8 + 3.8 + 3.8), which can be assigned to the Ti–OH nests caused by titanium vacancies. Notably, the chemical shift of the Ti–OH nests in TiO_2_/C_Inter_ moves to a high field,^35^ indicating that the interlayered carbon species have strong interactions with the Ti–OH nests. It can be deduced that low-temperature calcination in air could remove the surface carbon species and maintain the interlayered carbon, meanwhile contributing to the formation of titanium vacancies.

**Fig. 3:**
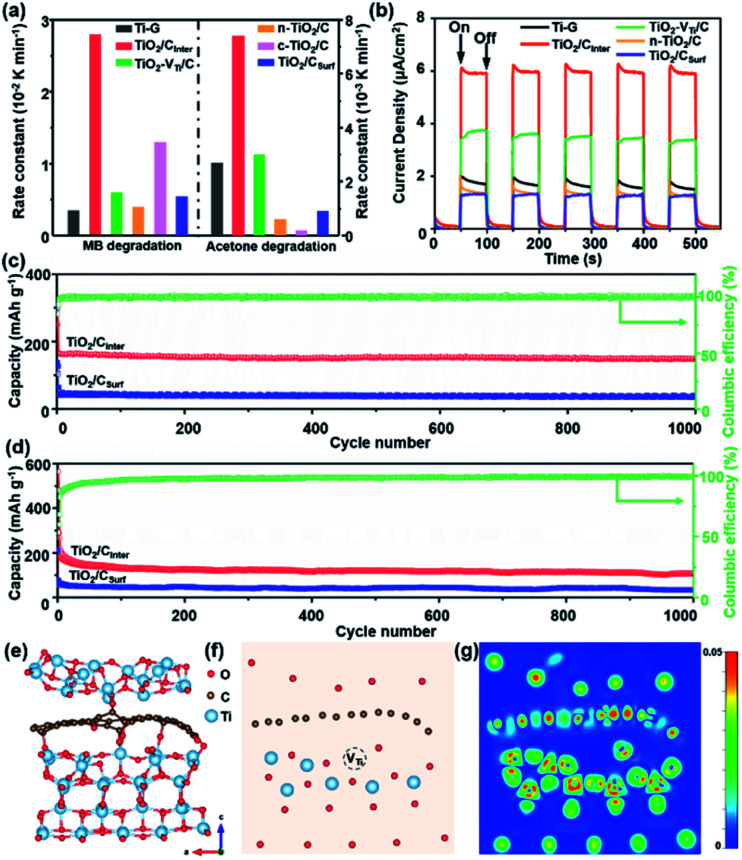
(a) Photocatalytic rate constants for degradation of methylene blue (MB) and acetone with (a) Ti-G, (b) TiO_2_/C_Inter_, (c) TiO_2_–V_Ti_/C, (d) n-TiO_2_/C (e) c-TiO_2_/C and (f) TiO_2_/C_Surf_, and (b) transient photocurrent response of Ti-G, TiO_2_/C_Inter_, TiO_2_–V_Ti_/C, n-TiO_2_/C and TiO_2_/C_Surf_. (c) Lithium-ion and (d) sodium-ion long cycle performance at a current density of 10 C. (e) The optimized model of TiO_2_ with interlayered carbon and titanium vacancies, (f) the section model of TiO_2_/C_Inter_ of the (010) facet and (g) the corresponding charge density difference.

To further investigate the role of interlayered carbon in TiO_2_/C_Inter_, photodegradation of an organic pollutant in liquid and gas phases (methylene blue, MB and acetone, respectively) was performed. For comparison, the performance of Ti-G, TiO_2_ with titanium vacancies and mixed carbon (named TiO_2_–V_Ti_/C), normal TiO_2_ without titanium vacancies but with carbon (named n-TiO_2_/C), commercial TiO_2_ nanotubes with carbon (named c-TiO_2_/C) and TiO_2_/C_Surf_ was also investigated. TiO_2_/C_Inter_ exhibits the highest photocatalytic activity (Fig. 3a) compared to other TiO_2_/C composites in MB and acetone degradation. The photocatalytic stability test of TiO_2_/C_Inter_ shows that around 97% of photocatalytic activity is retained after five cycles of photocatalysis (Fig. S15a, ESI†), and a strong signal of Ti-vacancies from EPR can be clearly observed (Fig. S15b, ESI†), suggesting the high stability of Ti-vacancies. Photoelectrochemical studies (Fig. 3b) reveal that TiO_2_/C_Inter_ exhibits the highest photocurrent intensity of 6 μA cm^−2^, which is 3-fold, 1.5-fold, 3.7-fold and 3.8-fold that of Ti-G, TiO_2_–V_Ti_/C, n-TiO_2_/C, and TiO_2_/C_Surf_, respectively. The EIS Nyquist plots of TiO_2_/C_Inter_ after irradiation with UV-Vis light display the smallest semicircle among all the samples (Fig. S16, ESI†), indicating that titanium vacancies and interlayered carbon are beneficial for the hole transfer to the electrolyte and thus greatly reduce the charge transfer resistance.^40^

TiO_2_/C samples were further applied as anode materials for lithium/sodium storage properties. It can be observed that TiO_2_/C_Inter_ exhibits the highest charge capacity after 300 cycles at 1 C in comparison with other TiO_2_/C anodes (Fig. S17, ESI†) and good structural stability (Fig. S18, detailed description in the ESI†). Further, TiO_2_/C_Inter_ has better reversible capacity and rate capability in comparison with TiO_2_/C_Surf_ (Fig. S19, ESI†). At a current density of 10 C (1.7 A g^−1^), TiO_2_/C_Inter_ shows a high reversible specific capacity of 148 mA h g^−1^ for lithium storage and 108 mA h g^−1^ for sodium storage after 1000 cycles at 10 C (Fig. 3c and d), demonstrating its outstanding cycling performances in comparison with other TiO_2_/C-based electrodes (Table S4, ESI†).

To gain further insight into the decisive role of interlayered carbon in efficient charge transfer and the stability of TiO_2_/C_Inter_ from a theoretical point of view, density functional theory (DFT) calculations were performed. The charge density difference of an optimized model of TiO_2_ with interlayered carbon and titanium vacancies (Fig. 3e and S20a, ESI†) shows obvious accumulation of electrons at the interfacial carbon layer in comparison with TiO_2_ with surface carbon species or no carbon (Fig. S20b–d, ESI†). The sectional charge density difference gives a clear view of the charge accumulation around the titanium vacancies and interlayer carbon, which could act as a bridge for efficient cascade and directed charge transfer from inside the lattice to the outer surface (Fig. 3f and g). The calculated formation energy (Tables S5 and S6†) indicates the good stability of TiO_2_ with interlayered carbon and titanium vacancies (detailed description of theoretical calculations in the ESI†). The calculation results clearly indicate that interlayered carbon can significantly facilitate the charge transfer and therefore enhance the photo/electro-catalytic performance.

**Fig. 4:**
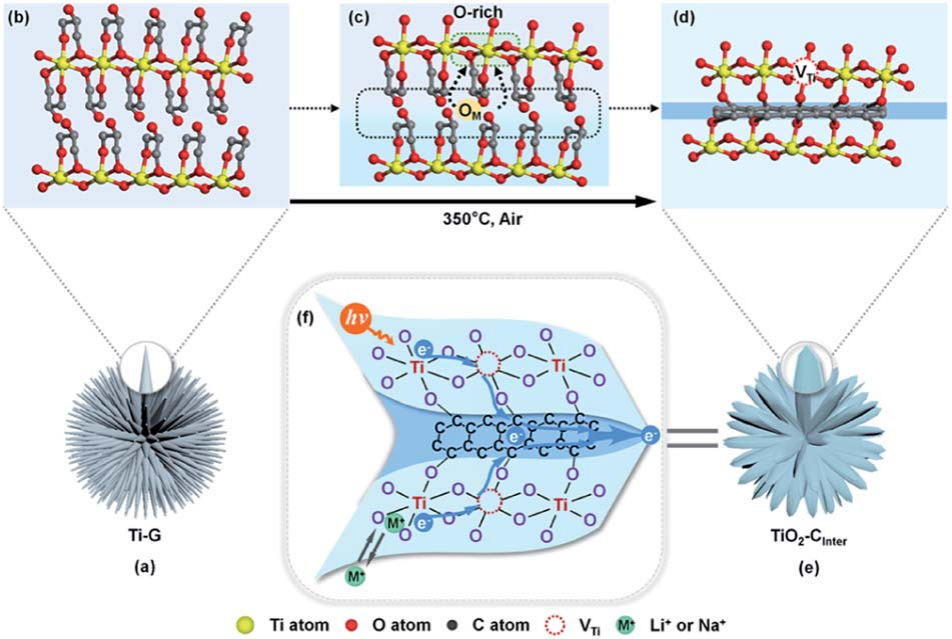
(a)–(e) Schematic illustration of the formation mechanism of TiO_2_/C_Inter_, (b–d) the simulated atomic change process from Ti-G to TiO_2_/C_Inter_, O_M_ refers to migrating oxygen. (f) Schematic description of the fast electron-transfer pathway and the proposed mechanism.

The formation process of TiO_2_/C_Inter_ we proposed is illustrated in Fig. 4. Ti-G with the oriented lamellar structure (Fig. 4a and b) starts to dehydrate and carbonize during the early stage of calcination at 350 °C. The surface carbon and inner carbon form, which contain sp^2^ and sp^3^ types of carbon. With prolongation of the calcination time, the surface carbon is oxidized and removed (Fig. 4c). At the same time, the inner carbon remains and further rearranges to the sp^2^ type because of the confinement effect by the lamellar structure of TiO_2_ (Fig. 4d).

In the meantime, the escaped oxygen from the inner phase would form an oxygen-rich environment at the interface, which is beneficial for the formation of titanium vacancies (Fig. 4c and d). The formation of titanium vacancies can be described as:
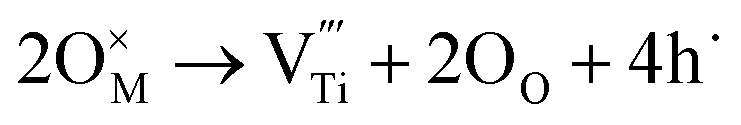
where 
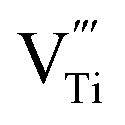
 represents a titanium vacancy, and h˙ represents a hole.

Finally, TiO_2_ nanosheets with interlayered carbon and titanium vacancies (Fig. 4d and e) are obtained by this low temperature calcination strategy (detailed description of the formation of interfacial defects in the ESI†).

The proposed mechanism of TiO_2_/C_Inter_ for photocatalysis and lithium/sodium storage is shown in Fig. 4f. The interlayer carbon and Ti-vacancies form a spatial inside-out electron transfer cascade from the lattice to the surface, which is not only beneficial to the charge separation in photocatalysis, but also enhances the interfacial conductivity for efficient electron transfer and Li^+^/Na^+^ insertion. Besides, the ultrathin nanosheet could shorten the diffusion length of electrons (including photogenerated electrons) and Li^+^/Na^+^, and the high specific area of nanosheets provides large contact area for photo/electrocatalytic reactions. Moreover, the amorphous TiO_2_/carbon interface is helpful to restrain the collapse of the nanostructure in the insertion/removal reactions, thus contributing to excellent stability.

## 3. Conclusions

In summary, TiO_2_ nanosheets with interfacial carbon and titanium vacancies have been prepared successfully by a controlled oxidation calcination. The confined carbon in the interlayer is crucial to a directional and efficient charge transfer, thus contributing to significantly increased photocatalytic activity and electrochemical performance. Our work provides a simple but effective way for high-performance design of semiconductors featuring low cost, high efficiency, and high stability.

## Data availability

The data that supports the findings of this study are available within the ESI† and from the corresponding author upon reasonable request.

## Author contributions

S. M. W. carried out the experiments of synthesis, photocatalytic performance and the lithium/sodium storage experiments. X. Y. Y. conceived the project, provided the idea, and guided the experiments. Y. T. W., Y. X. Z. S. T. X. helped with the experiments. X. F. Z. and L. Y. W. helped with the NMR measurements and corresponding analysis. S. M. W. and X. Y. Y. proposed the mechanisms. J. B. C. performed the DFT calculation and helped with the analysis. G. T. performed the measurements of TEM. S. M. W. and X. Y. Y. wrote and revised the paper. C. J., M. S. and D. W. B. revised the paper. All authors have given approval to the final version of the manuscript.

## Conflicts of interest

There are no conflicts to declare.

## Supplementary Material

SC-013-D2SC01872A-s001

SC-013-D2SC01872A-s002
