# Supplementary material for: Design and synthesis of TiO2/C nanosheets with a directional cascade carrier transfer
Source: Chem Sci. 2022 May 10;13(24):7126–31. doi: 10.1039/d2sc01872a (PMC9214889; doi:10.1039/d2sc01872a)
Supplement: SC-013-D2SC01872A-s002 [file SC-013-D2SC01872A-s002.pdf]

Electronic Supplementary Material (ESI) for Chemical Science.
This journal is © The Royal Society of Chemistry 2022

Supporting Information

Design and synthesis of TiO_2/C nanosheets with a directional cascade carriers transfer

*Si-Ming Wu, Yi-Tian Wang, Shi-Tian Xiao, Yan-Xiang Zhang, Ge Tian, Jiang-Bo Chen, Xiao-Fang Zhao, Christoph Janiak, Menny Shalom, Detlef W. Bahnemann, Li-Ying Wang, Xiao-Yu Yang**

Experimental Procedures

1. Materials

Tetrabutoxytitanium ($Ti(OC_4H_9)_4$, TBOT) and glycerol was purchased from Aladdin. Ethanol was purchased from Shanghai Chemical Regent Factory of China. Commercial TiO_2 nanotubes (designed as comm-TiO_2) were purchased from DX nano. Co of China. Deionized water was used in all experiments.

2. Preparation of Ti-G, TiO_2/C_{Inter} and TiO_2/C_{Surf}.

Typically, 1 mL TBOT was added into 10 mL glycerol and 30 mL ethanol while stirring. The mixture was transferred into a 100 mL Teflon-lined stainless-steel autoclave and put in an oven with 180°C for 24h. The obtained product was filtered and washed with ethanol, and then dried at 60 °C overnight. The powers are referred as Ti-G. After calcined at 350 °C for 4 h with a heating rate to the target temperature of 1 °C/min, grey sample is obtained and designed as TiO_2/C_{Inter}. As comparison, the sample calcined at 350 °C for 4 h under Argon atmosphere are also prepared and designed as TiO_2/C_{Surf}.

3. Preparation of TiO_2-V_{Ti}/C, n-TiO_2/C and comm-TiO_2/C.

To prepare TiO_2-V_{Ti}/C and n-TiO_2/C, TiO_2-V_{Ti} was firstly prepared by the calcination of As-TiO_2 at 550 °C for 4 h with a heating rate to the target temperature of 1 °C/min. n-TiO_2 was prepared by the calcination of Ti-G at 750 °C for 4 h with a heating rate to the target temperature of 1 °C/min. Then TiO_2-V_{Ti}/C, n-TiO_2/C and comm-TiO_2/C were prepared by mixing amorphous carbon and the TiO_2 sample respectively, with further grinding process.

4. Characterization

Thermogravimetric analysis (TGA) was conducted using a NETZSCH STA 449 F3 thermogravimetric analyzer with a heating rate of 5 °C min^{-1} under air. Scanning electron microscopy (SEM) images were taken with an S4800 electron microscope operating at 5 kV

to observe the morphology. Powder X-ray diffraction (XRD) patterns were recorded by a D8 Advance X-ray diffractometer (Bruker, Germany) with Cu-Kα radiation (λ = 0.15406 nm) operated at 40 kV, 40 mA. The nitrogen adsorption and desorption isotherms of the samples were measured using a Micromeritics ASAP 2020M system. Before the measurements, the samples were outgassed at 100 °C in vacuum for 12 h. The BET surface area was determined by a multipoint BET method using the adsorption data in a relative pressure (P/P_0) range of 0.05–0.3. The Barrett-Joyner-Halenda (BJH) method was used to determine the pore sizes. Raman analysis was performed using a Raman spectrometer (LabRam HR Evolution, Horiba, France) under visible excitation at 532 nm. Fourier-transform infrared (FT-IR) spectra were obtained with a Thermo Nicolet 360 spectrometer. Transmission electron microscopy (TEM) experiments were conducted on a Talos-F200S STEM/EDS electron microscope operated at 200 kV. Atomic force microscopy (AFM) measurements were performed on a Dimension FastScan Microscope (Bruker, Germany). Electron paramagnetic resonance (EPR) measurements were performed at the X-band using a JEOL FA 2000 spectrometer. The microwave frequency was 9.163 GHz, the modulation amplitude was 0.1 mT, the microwave power was 1 mW, and the experimental temperature was 295 K. Ultraviolet-visible spectroscopy diffuse reflectance spectra (UV-vis DRS) were attained using a Shimadzu UV-vis spectrophotometer (UV-2550). The spectra from 200 to 800 nm were taken at room temperature in air. X-ray photoelectron spectra (XPS) of the samples were recorded on a PHI Quantera II, (ULVAC-PHI, Japan) using a monochromated Al-Kα X-ray source. Accurate binding energies (±0.1 eV) were determined with respect to the position of the adventitious C 1s peak at 284.8 eV. ^{13}C cross-polarization/magic-angle spinning nuclear magnetic resonance (CP/MAS NMR) experiments were carried out in a 7 mm MAS probe on a Bruker AVANCE-III 400 spectrometer with a sample spinning rate of 5 kHz, a ^{13}C $\pi/2$ pulse length of 5 μs and a recycle delay of 1 s.^{1}H MAS and 2D ^{1}H TQ-SQ MAS NMR spectra were carried out in a 1.9

mm MAS probe on a Bruker AVANCE-III 500 spectrometer with a sample spinning rate of 38 kHz, a ^{1}H $\pi/2$ pulse length of 1.65 μs and a recycle delay of 2 s.

5. Photocatalytic Experiment.

To investigate the photocatalytic activity of the nanostructured TiO_2 samples, methylene blue (MB) dye was used for photodegradation. Typically, 20 mg photocatalysts were dispersed in 100 mL aqueous solution containing 3×10^{-5} mol/L MB. For photocatalysis, the samples were irradiated under UV-vis light with a PLS-SXE-300D lamp (Beijing Perfectlight Technology Co., Ltd.). The photodegradation of MB was monitored by UV-visible spectrometry (UV2550, Shimadzu, Japan).

Acetone was used as a model air pollutant to measure the photodegradation ability of the TiO_2 samples obtained. The TiO_2 photocatalysts were dispersed in aqueous solution and then dispersed onto a dish with a diameter of ca. 3 cm. The dish was then dried at 80 °C for 12 h and then cooled to room temperature before being used. The weight of each catalyst was kept at 100 mg. After putting the photocatalysts into the reactor, 5 μL of acetone was injected into the reactor with a micro syringe. The reactor was kept in the dark for a certain time to reach adsorption-desorption equilibrium before irradiation. The analysis was conducted with a gas chromatograph (Agilent 2920B) equipped with a flame ionization detector (FID).

6. Electrochemical Measurements.

Electrochemical tests were performed using 2025-type coin cells. The working electrodes were synthesized by mixing the active materials, acetylene black, and poly (vinylidene fluoride) (PVDF) with a weight ratio of 7:2:1 in N-methyl-2-pyrrolidone (NMP) to form a slurry. The slurry was uniformly spread on a copper foil. Lithium foil was used as the counter electrode and reference electrode. A 1 mol/L solution of $LiPF_6$ dissolved in ethylene carbonate and dimethyl carbonate (1:1 in volume ratio) was used as the electrolyte. Na half-

cells were assembled with a Na metal foil as the negative electrode and 1 mol/L $NaClO_4$ in ethylene carbonate and dimethyl carbonate (1:1 in volume ratio) as the electrolyte. All the half-cells were assembled in an argon-filled glovebox with both water and oxygen contents below 0.5 ppm. Galvanostatic discharge-charge curves were collected on a LAND CT2001A battery test system within a voltage range of 3.0-1.0 V (vs Li^+/Li) and 3.0-0.1V (vs Na^+/Na) at 1 C rate kept after first three cycles (C/5) (1 C is defined as 170 mA/g). All electrochemical measurements were carried out at 25 °C. Electrochemical impedance spectra (EIS) measurements were carried out on an electrochemical workstation (Autolab PGSTAT302N, Metrohm, Switzerland) in a frequency range of 0.1 MHz to 0.01 Hz.

7. Photo-electrochemical Measurements.

Photocurrent tests were carried out in a conventional three-electrode system using on a Autolab PGSTAT302N electrochemical workstation (Metrohm, Switzerland) with a Pt foil as the counter electrode and a Ag/AgCl reference electrode under a PLS-SXE-300D lamp. The working electrodes were prepared by dispersing catalysts (5 mg) and Nafion solution (100 μL, 0.5 wt%) in water/ethanol mixed solvent (1 mL, 1:1 v/v) at least 30 min of sonication to form a homogeneous ink. The working electrode was synthesized by drop-casting the above ink (40 μL) onto FTO glass with an area of 1 cm^2.

8. Density Functional Theory Calculations.

The generalized gradient approximation (GGA) with the Perdew-Burke-Ernzerhof exchange correlation functional (adding Grimme method for DFT-D3 correction were performed to study the formation mechanism of Ti-vacancy) and a 520 eV cutoff for the plane-wave basis set were employed to perform all the density functional theory (DFT) computations of the materials within the frame of Vienna ab initio simulation package (VASP). The convergence threshold was set as 10^{-5} eV in energy and 0.02 eV/Å in force. 1×3×1 Monkhorst-Pack grid k-points were employed for geometric optimization. In this study, the C are represented by C42

and the anatase TiO_2 (001) surface is represented by the anatase TiO_2 (001) surface model. The model of TiO_2-V_{Ti}-interlayered carbon is constructed by a periodically repeated four-layer 2×5 slab of atoms(down) (including titanium vacancy), one layer of carbon and two-layer 2×5 slab of atoms(up) including 49 Ti atoms, 100 O atoms and 42 C atoms. The model of TiO_2-V_{Ti} is constructed by a periodically repeated four-layer 2×5 slab of atoms including 33 Ti atoms and 72 O atoms. The model of TiO_2-V_{Ti}-surface carbon is constructed by a periodically repeated four-layer 2×5 slab of atoms and one layer of carbon atoms including 33 Ti atoms, 72 O atoms and 42 C atoms. All layer is relaxed with a vacuum region of approximately 30 Å.

Results and Discussion

Table S1. The effects of calcination temperatures on the physicochemical properties of the TiO_2 samples.

Calcination temperature	Calcination atmosphere	S_{BET} (m^2 g^{-1})	Pore volume (cm^3 g^{-1})	Pore Diameter (nm)
As-prepared	/	54	0.25	4.9
350	Air	110	0.31	2.7
550	Air	36	0.19	2.4
750	Air	6	0.01	N/A
350	Ar	24	0.08	N/A

Table S2. The fitted peak area and calculated *sp^2/sp^3* ratio of TiO_2/C_{Inter} and TiO_2/C_{Surf} from ^{13}C NMR.

Sample	Peak	$^aS_{sp2}$				$^bS_{sp3}$		$^c sp^2/sp^3$ ratio
TiO_2/C_{Inter}	Location (ppm)	187.6	180.0	167.7	136.6	36.0	26.1	6.67
	Area	7.57E6	1.15E7	1.20E6	2.15E7	4.59E6	1.67E6	
TiO_2/C_{Surf}	Location (ppm)	132.3				29.3		2.50
	Area	1.11E7				4.43E6		

[a] The peak area of *sp^2* carbon is the total amount of the fitted peaks in S_{sp2} in Fig. S9.
[b] The peak area of *sp^3* carbon is the total amount of the fitted peaks in S_{sp3} in Fig. S9.
[c] It has to be pointed out that the numerical values of the calculated *sp^2/sp^3* ratio here don't mean the exact amount of the *sp^2/sp^3* ratio in the samples. They can be regarded as reference for the comparison of their relative amount in TiO_2/C_{Inter} and TiO_2/C_{Surf}.

Table S3. The peak area and molar ratio on the surface of different TiO_2 samples.

Sample	Calcination condition	[a]Lattice Ti	[b]Lattice O	[c]Carbon	[d]Surface O/Ti ratio	[e]Surface C/Ti ratio
Ti-G	-	84620	179211	75519	5.44±0.1	6.42±0.1
TiO_2/C_{Inter}	350-air	213750	194796	20626	2.34±0.1	0.69±0.1
TiO_2-V_{Ti}	550-air	196112	165147	18967	2.16±0.1	0.69±0.1
n-TiO_2	750-air	151473	118646	18676	2.00±0.1	0.88±0.1
TiO_2/C_{Surf}	350-Ar	175168	164582	76025	2.45±0.1	3.12±0.1

[a] The peak area of lattice Ti is the combination of Ti $2p_{3/2}$ and Ti $2p_{1/2}$ XPS peaks.
[b] The peak area of lattice O is the combination of O^{2-}, O^- and C-O peaks.
[c] The peak area of carbon is the total amount of C 1s peaks.
[d,e] The calculation of surface O/Ti ratio and surface C/Ti ratio is based on the formula as following:

$$n_a:n_b=I_a/S_a: I_b/S_b$$

S is relative sensitivity factor, which is 2.001 for Ti element, 0.78 for O element and 0.278 for C element. I is the peak area.

Table S4. Properties and electrochemical performance of carbon incorporated TiO_2 as negative electrode materials for lithium/sodium ion batteries.

Materials	Lithium storage cycling capacity (mAh g^{-1}) 1 C=170 mA g^{-1}	Sodium storage cycling capacity (mAh g^{-1}) 1 C=170 mA g^{-1}	Ref
TiO_2/C_{Inter}	148 after 1000 cycles at 10 C<br>165 after 300 cycles at 1C	108 after 1000 cycles at 10 C<br>135 after 300 cycles at 1C	Our work
NS-HCNF@S-TiO_2	153 after 300 cycles at 1 A g^{-1}	120 after 300 cycles at 1 A g^{-1}	1
TiO_2@RGO	106 after 50 cycles at 2 A g^{-1}	112 after 50 cycles at 1 A g^{-1}	2
$TiO_{2-x}C_x$	91.5 after 1000 cycles at 10 C	-	3
TiO_2/RGO	130.6 after 1000 cycles at 10 C	-	4
TiO_2 QDs/Graphene NS	147 after 100 cycles at 10C	-	5
TiO_{2-x}/CNT	158 after 1000 cycles at 1 A g^{-1}	-	6
TiO_2-NCF	149 after 100 cycles at 1 A g^{-1}	-	7
TiO_2/CNT	129 after 1000 cycles at 10 C	-	8
TiO_2@C	-	101 after 1000 cycles at 1 A g^{-1}	9
NC@TiO_2/TiF_3	-	63 after 1000 cycles at 1 A g^{-1}	10
N-doped TiO_2/C	-	121 after 200 cycles at 1C	11
TiO_2/CMFs		104 after 500 cycles at 0.5 A g^{-1}	12

Table S5. The calculated formation energy of different models.

Model	E_{total} (eV)	E_C (eV)	$E_{TiO2(down)}$ (eV)	$E_{TiO2(up)}$ (eV)	^aE_f (eV)
TiO_2-V_{Ti}-surface carbon	-1271.5	-350.6	-884.1	-	-36.8
TiO_2-V_{Ti}-interlayered carbon	-1655.7	-345.1	-884.9	-376.7	-45

[a]The calculation of formation energy (E_f) of different TiO_2/C model is based on the formula listed below:

$$E_f = E_{total} - E_C - E_{TiO2}$$

where E_{total} is the total energy of the model, E_C is the energy of carbon layer, and E_{TiO2} is the energy of TiO_2.[13]

For TiO_2/C_{Surf}, E_f=E_{total} - E_C - $E_{TiO2(down)}$ and for TiO_2/C_{Inter}, E_f=E_{total} - E_C - $E_{TiO2(down)}$ - $E_{TiO2(up)}$.

Table S6. The calculated formation energy of titanium vacancies.

Model	TiO_2	TiO_2-surface carbon	TiO_2-interlayered carbon	TiO_2-V_{Ti}	TiO_2-V_{Ti}-surface carbon	TiO_2-V_{Ti}-interlayered carbon
E_{total} (eV)	-921.35	-1289.13	-1672.36	-903.96	-1271.50	-1655.71
[a]E_V (eV)	-	-	-	11.87	12.11	11.13

[a]The calculation of formation energy of Ti vacancies (E_v) is based on the formula listed below:

$$E_v = E_{total}^{defect} - E_{total}^{host} + \mu_{Ti}^{FERE}$$

where E_{total}^{defect} and E_{total}^{host} host are the total energies of the models with and without Ti vacancies, respectively, and μ_{Ti}^{FERE} is the standard state Ti chemical potential, the value of μ_{Ti}^{FERE} is -5.52 eV.[14-15]

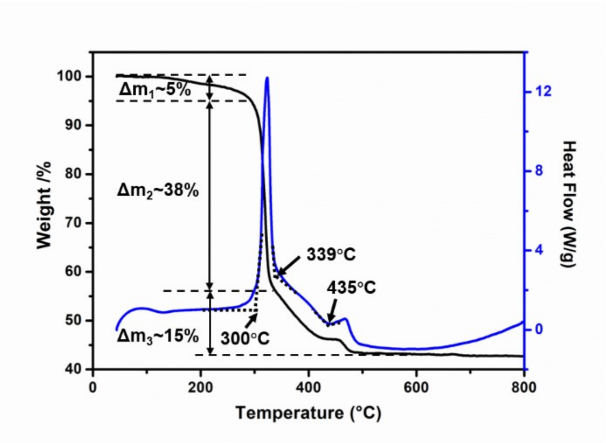

Fig. S1. TG and DTA curves of Ti-G.

Detailed descriptions of TGA-DTA.

Thermogravimetric analysis-differential thermal analysis (Fig. S1) of titanium glycerolate (designated as Ti-G) was used to determine the calcination profile. The slight weight loss below 300 °C is due to the loss of absorbed water, the weight loss of 38% in the range of 300-350 °C is due to the dehydroxylation process and combustion of unstable carbon on the surface. A phase transformation temperature of around 300 °C indicates the transformation of the precursor to anatase TiO_2. 435 °C is phase transformation temperature from anatase to rutile structure. After 500 °C, the carbon species in the TiO_2 is totally removed and there is no mass change with further calcination. Therefore, 350 °C is chosen for the synthesis of metastable anatase phase TiO_2/C composite without surface carbon and with the remaining interlayered carbon. The loss of internal carbon starts at about 339 °C and ends at about 500 °C as indicated by the DTA curve. According to TGA curve, the weight loss of interlayered carbon is 15 wt% compared with the Ti-G, and the calculated amount of interlayered carbon is 26.3 wt% in TiO_2/C_{Inter}.

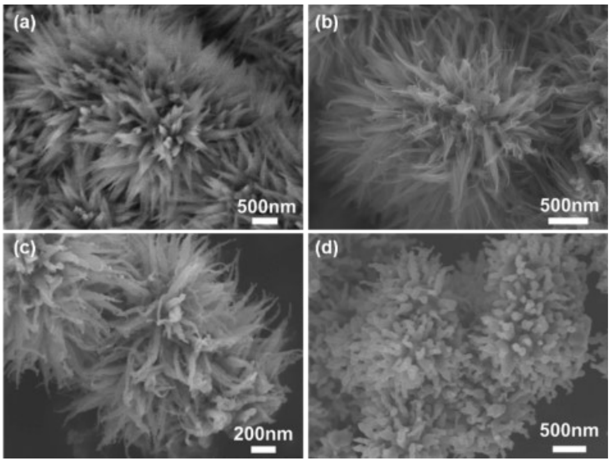

Fig. S2. SEM images of (a) Ti-G, and Ti-G calcined at (b) 350°C, (c) 550 °C and (d) 750 °C.

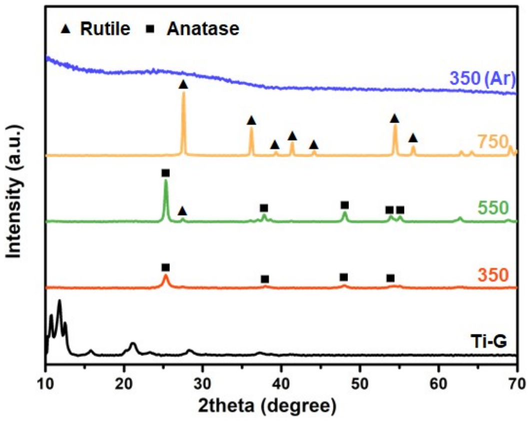

Fig. S3. XRD patterns of a) Ti-G and Ti-G calcined at 350°C, 550 °C, 750 °C in air and 350°C in Ar.

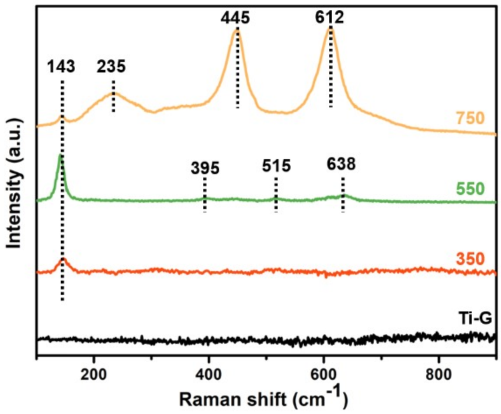

Fig. S4. Raman spectra of a) Ti-G and Ti-G calcined at 350°C, 550 °C and 750 °C. with the excitation line at 532 nm. The peaks at 143, 395, 515, and 638 cm^{-1} could be belonged to typical anatase Raman bands of E_g, B_{1g}, $A_{1g}(B_{1g})$, and E_g modes, respectively. The peaks at 235, 445 and 612 cm^{-1} could be assigned to the typical rutile Raman bands of B_{1g}, E_g and A_{1g} modes, respectively.[16]

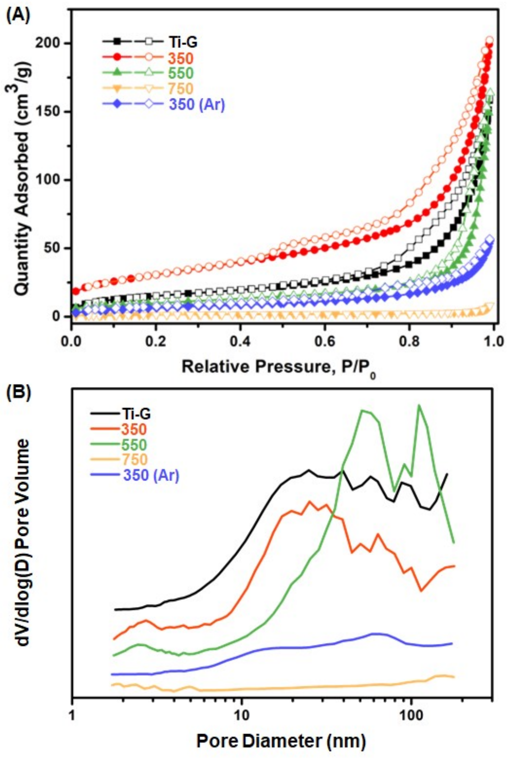

Fig. S5. (A) Nitrogen-adsorption-desorption isotherms and (B) corresponding pore size distribution of a) Ti-G and Ti-G calcined at 350°C, 550 °C, 750 °C in air and 350°C in Ar.

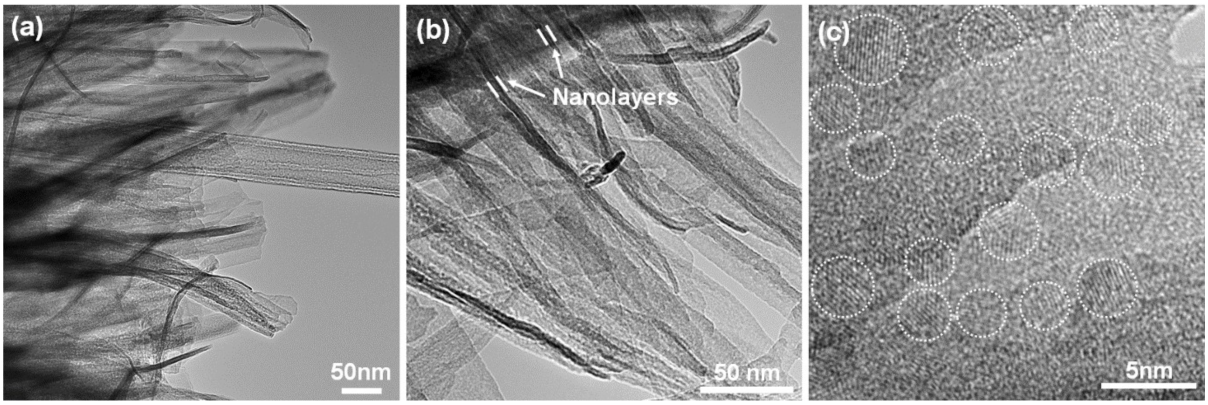

Fig. S6. (a), (b) TEM image and (c) Magnified TEM image of TiO_2/C_{Inter}. Representative TiO_2 nanocrystals are marked with circles.

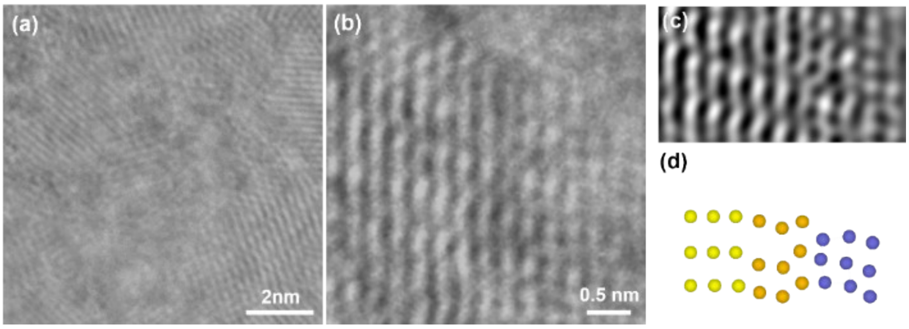

Fig. S7. (a) Original TEM image in Fig. 1h; Original (b) TEM image (c) Inverse FFT image and (d) atomic models in Fig. 1i.

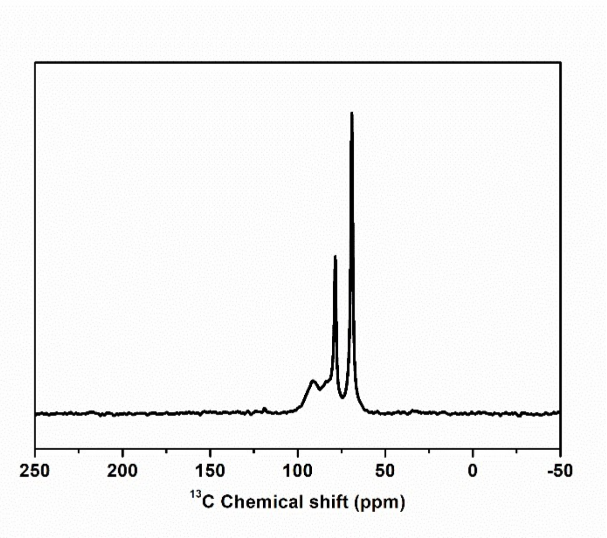

Fig. S8. ^{13}C NMR spectrum of Ti-G.

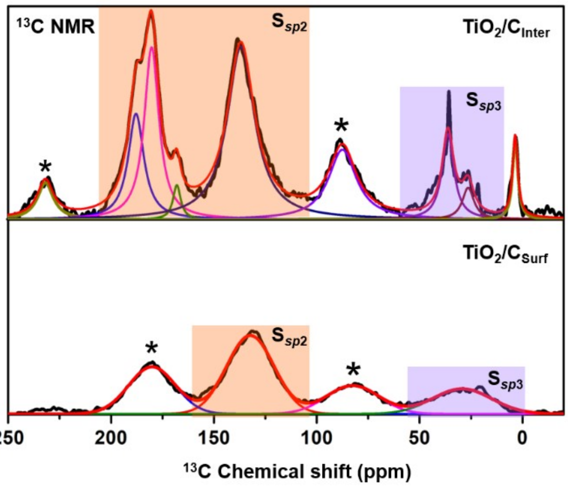

Fig. S9. The fitted ^{13}C NMR spectra of TiO_2/C_{Inter} and TiO_2/C_{Surf}, * indicates the signal of rotor cap. The ratio of sp^2 and sp^3 in each sample is obtained by the calculated ratio of fitted peak areas in S_{sp2} and S_{sp3}. The detailed data is shown in Table S2.

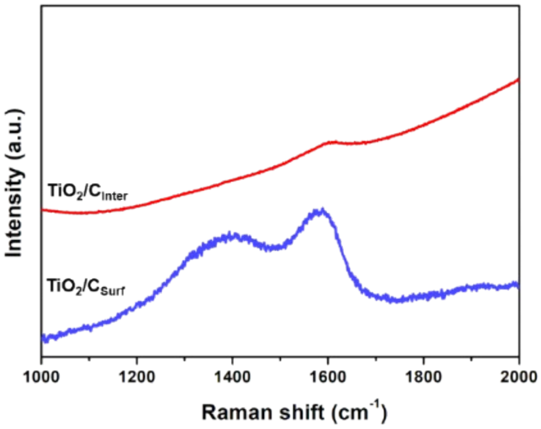

Fig. S10. Raman spectra of TiO_2/C_{Inter} and TiO_2/C_{Surf}.

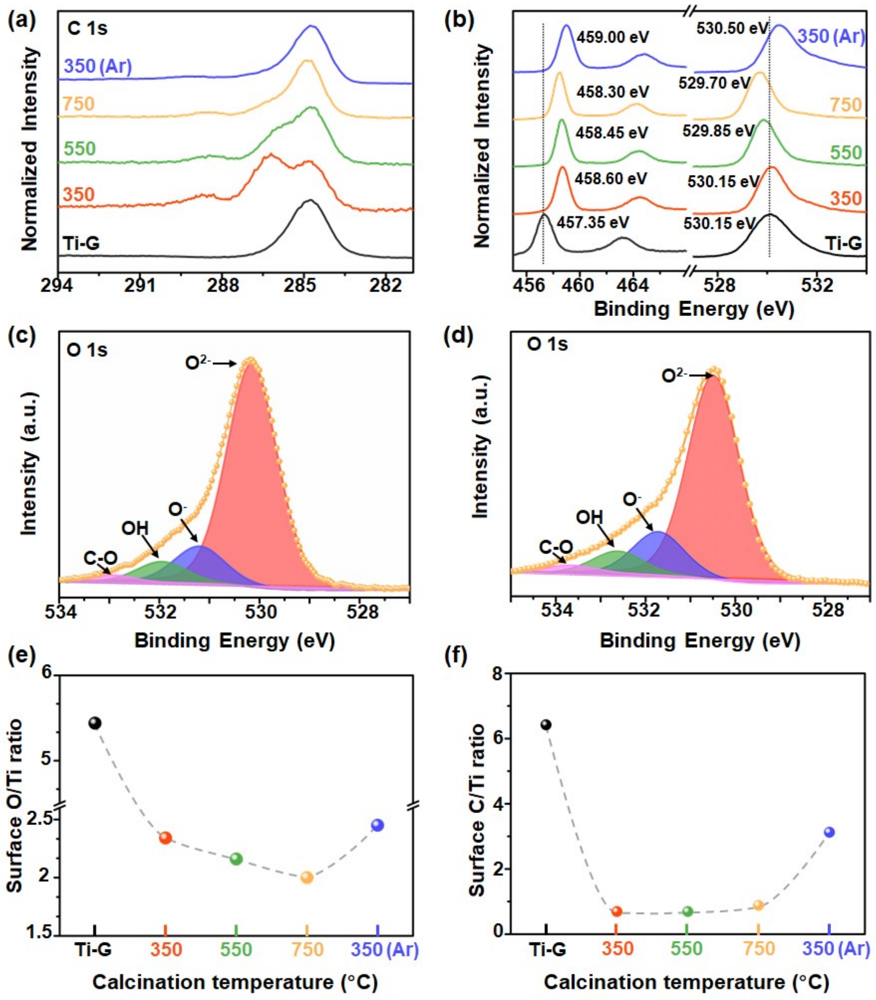

Fig. S11. (a) C 1s XPS spectra, (b) Ti 2p and O 1s XPS spectra of a. Ti-G, b. TiO_2/C_{Inter}, c. Ti-G calcined at 550 °C, d. Ti-G calcined at 750 °C and e. TiO_2/C_{Surf}, (c) fitted O 1s peaks of TiO_2/C_{Inter}, (d) fitted O 1s peaks of TiO_2/C_{Surf}, (e) the calculated surface O/Ti molar ratio and (f) surface C/Ti molar ratio of TiO_2 samples calcined at different temperature.

Detailed descriptions of XPS.

XPS of TiO_2/C_{Inter} and TiO_2/C_{Surf} are recorded for further investigation (Fig. 2b, Fig. S11a, b). The O1s core-level XPS of TiO_2/C_{Inter} (Fig. S11b) display the major peaks caused by the O^{2-} ions in the O-Ti-O lattice (around 530.0 eV).[17] The apparent shoulder peak is fitted by three peaks, which are attributed to the OH groups on the surface (around 532.0 eV), O^{1-} species (around 531.2 eV) and C-O species (533.0 eV), respectively.[18,19] The O^- species are usually caused by Ti vacancies, and the C-O species may attribute to the C-O-Ti bond formed after

the calcination process. Similar oxygen species are observed in TiO_2/C_{Surf} with positive chemical shift of each peak owing to the different chemical environment of TiO_2/C_{Inter} and TiO_2/C_{Surf}. In comparison with the Ti $2p_{3/2}$ XPS spectra of Ti-G, TiO_2/C_{Inter}, the sample calcined at 550 °C, the sample calcined at 750 °C and TiO_2/C_{Surf} show a chemical shift of 1.25 eV, 1.10 eV, 0.95 eV and 1.65 eV respectively (Fig. S11b). The Ti-O-C bond will lead to a higher electron cloud density and thus result in positive Ti 2p core level shifts. In our cases, these Ti 2p core level shifts occurred in all composite samples mainly due to the generated Ti-O-C bonds and the phase transformation of TiO_2.

Surface O/Ti ratio. The surface O/Ti atomic ratio can be calculated from the Ti 2p and O 1s peaks of the samples as reference data to analysis the change of the structure. The O/Ti atomic ratio is calculated to be 5.44±0.1 for Ti-G, 2.34±0.1 for TiO_2/C_{Inter}, 2.16±0.1 for the sample calcined at 550 °C, 2.00±0.1 for the sample calcined at 750 °C and 2.45±0.1 for TiO_2/C_{Surf} (Fig. S11d and Table S2), indicating the oxygen-rich environment provided by the precursor and the titanium vacancies in TiO_2/C_{Inter}.

Surface C/Ti ratio and *Surface C content*. The surface C/Ti atomic ratio can be calculated from the Ti 2p and C 1s peaks of the samples (Fig. S11a, f, Table S2). The C/Ti atomic ratio is calculated to be 6.42±0.1 for Ti-G, 0.69±0.1 for TiO_2/C_{Inter}, 0.69 for the sample calcined at 550 °C, 0.88±0.1 for the sample calcined at 750 °C and 3.12±0.1 for TiO_2/C_{Surf} (Fig. S11d and Table S2). It is obvious that the C/Ti ratio of TiO_2/C_{Inter} is similar with the samples without carbon by calcining at higher temperature, which indicates that the amount of surface carbon species in these samples are almost the same. Similar results are also obtained from the atomic amount of carbon by XPS, which is 47.0% for Ti-G, 15.0% for TiO_2/C_{Inter}, 15.5% for TiO_2-550, 19.5% for TiO_2-750 and 44.7% for TiO_2/C_{Surf}. It has to be clarified that the C content contains not only the carbon species in the structure but also the absorbed carbon species, so the numeric value is not applicable. But on the premise that all samples have the similar amount of absorbed carbon species, then a big difference is observed between the sample calcined in air and inert atmosphere. A large amount of surface carbon has been removed in TiO_2/C_{Inter} while most of the carbon species are retained in TiO_2/C_{Surf}. This is in accordance with our previous analysis that calcination in inert atmosphere lead to over-carbonization. And it can also clarify that the carbon species in TiO_2/C_{Inter} are mostly interlayered carbon instead of surface carbon.

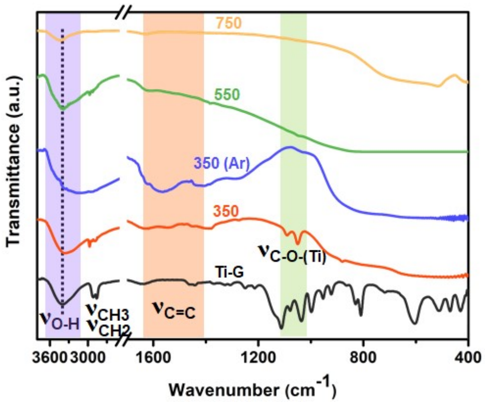

Fig. S12. FT-IR spectra of a. Ti-G, b. TiO_2/C_{Inter}, c. TiO_2/C_{Surf}. d. Ti-G calcined at 550 °C, e. Ti-G calcined at 750 °C.

Detailed descriptions of FT-IR

For Ti-G, the band at 3400 cm^{-1} corresponds to the O-H stretching mode. The strong absorption bands in the 2500-3000 cm^{-1} domain are characteristics of the C-H mode. The bands between 1400-1650 cm^{-1} are due to the vibration of C=C. The bands located at 1113, 1079, 1037 cm^{-1} are assigned to the butoxy groups linked to titanium. [20, 21] After calcination at 350 °C in air, most of these absorption bands disappeared in TiO_2/C_{Inter}, with two remaining bands at 1091 and 1050 cm^{-1} assigned to the Ti-O-C bonds. With further increasing of the calcination temperature, highly crystallized TiO_2 is obtained, resulting in the disappearance of the Ti-O-C bond. In contrast, calcination at 350 °C in Ar would lead to the transformation of C-H groups to the C=C groups, which mostly exist in the surface amorphous carbon.

The peaks assigned to the organic functional group of Ti-G disappear in TiO_2/C_{Inter}, and the peak of C-O-Ti bond and hydroxyl groups remain, indicating the removal of most surface carbon and the formation of inner carbon. With increasing calcination temperature, the C-O-Ti bond and hydroxyl group disappear, indicating the increase of crystallinity and the complete removal of carbon. Therefore, it can be deduced that most of the surface carbon

species are removed in TiO_2/C_{Inter}, and the remaining interlayered carbon form the C-O-Ti bonds with the TiO_2 lattice.

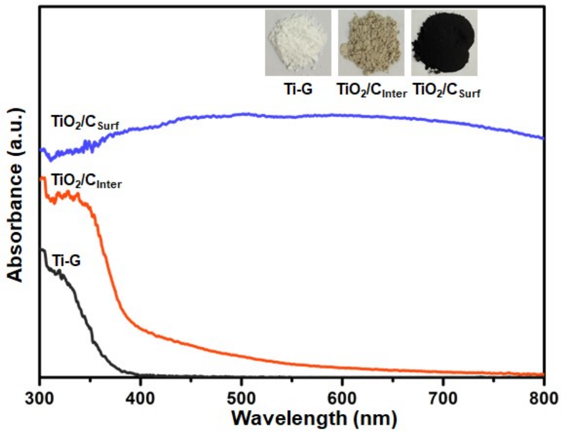

Fig. S13. UV-vis DRS spectra of Ti-G, TiO_2/C_{Inter} and TiO_2/C_{Surf}, inset are the corresponding optical photographs.

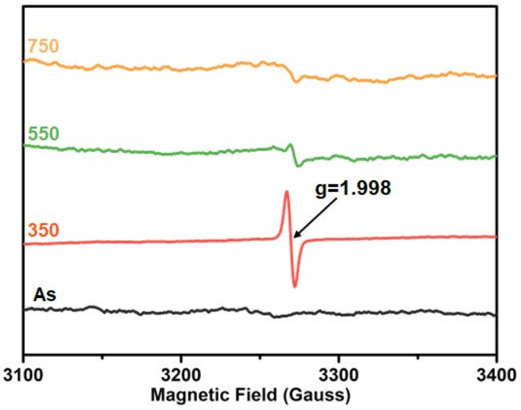

Fig. S14. EPR spectra of Ti-G, Ti-G calcined at 350 °C (TiO_2/C_{Inter}), 550 °C and 750 °C.

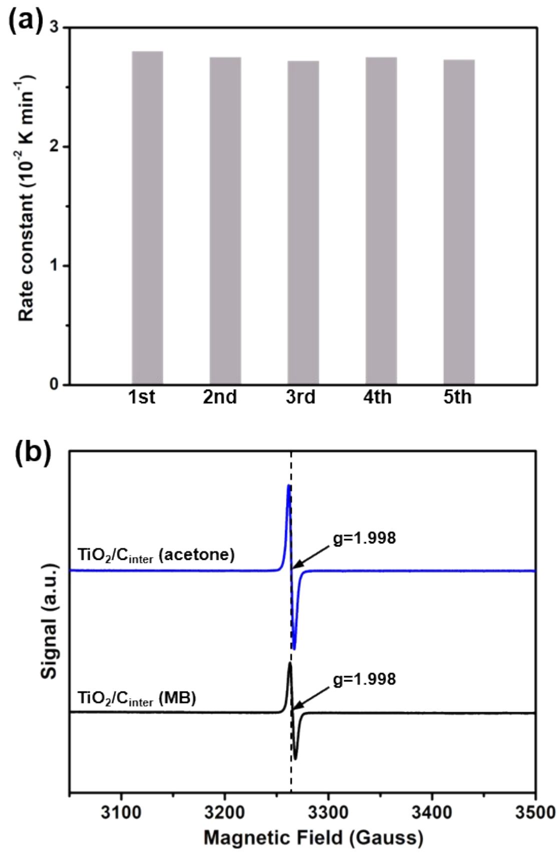

Fig. S15. (a) Photocatalytic stability test for methylene blue (MB) photodegradation under UV irradiation over TiO_2/C_{Inter}. (b) EPR spectra of TiO_2/C_{Inter} after MB photodegradation and acetone photodegradation.

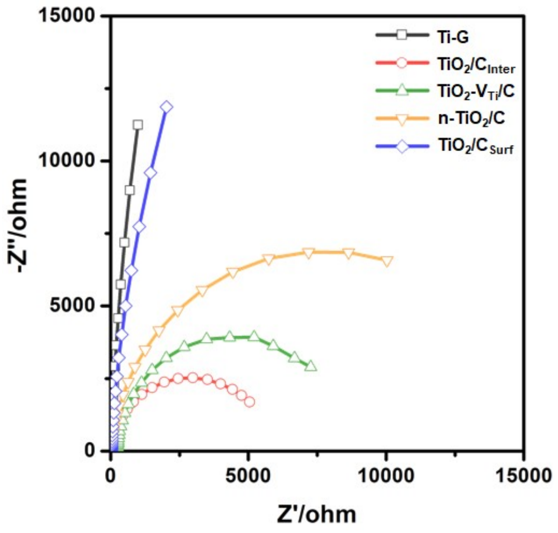

Fig. S16. EIS Nyquist plots of a) Ti-G, b) TiO_2/C_{Inter}, c) TiO_2-V_{Ti}/C, d) n-TiO_2/C and e) TiO_2/C_{Surf} collected at open circuit potential under irradiation of UV-vis light.

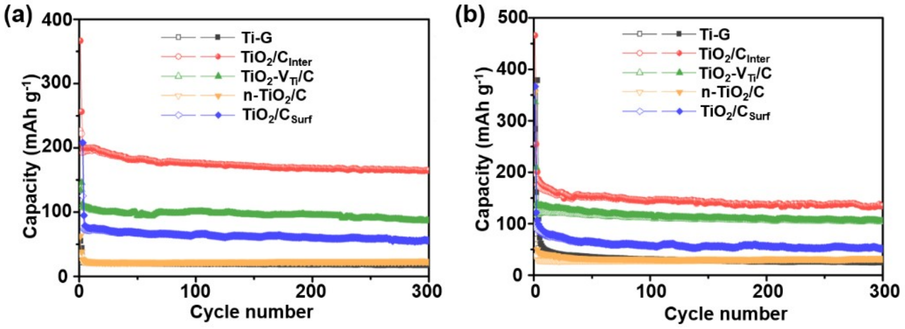

Fig. S17. (a) Lithium-ion and (b) Sodium-ion cycle performances of Ti-G, TiO_2/C_{Inter}, TiO_2-V_{Ti}/C, n-TiO_2/C and TiO_2/C_{Surf} at a current density of 1 C.

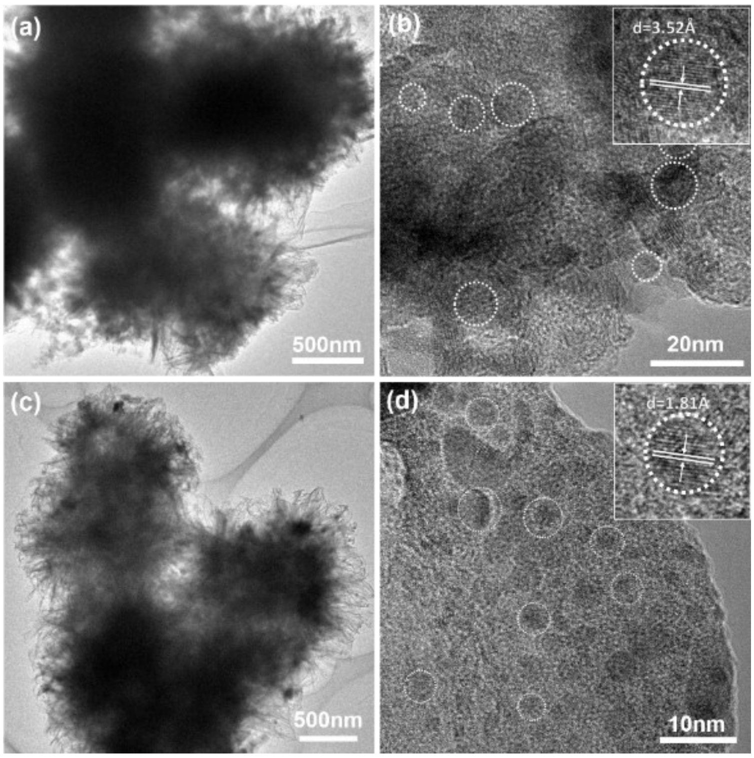

Fig. S18. (a) TEM image and (b) HRTEM image of TiO_2/C_{Inter} after the lithium-ion cycling performance for 300 cycles and corresponding $Li_2Ti_2O_4$ nanoparticle (inset image); (c) TEM images and (b) HRTEM image of TiO_2/C_{Inter} after the sodium-ion cycling performance for 300 cycles and corresponding Na_xTiO_2 nanoparticle (inset image).

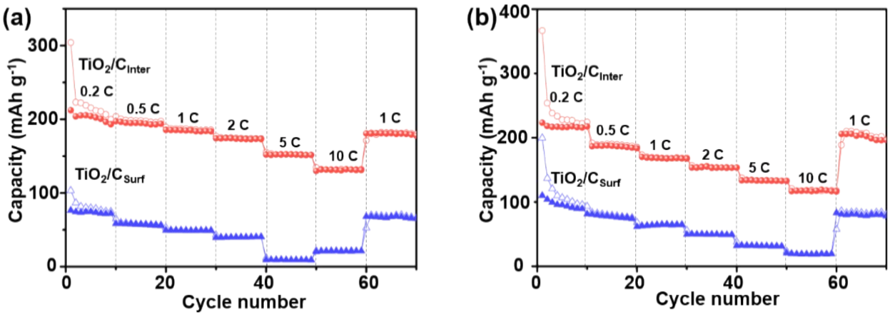

Fig. S19. Comparison of the rate performance of TiO_2/C_{Inter} and TiO_2/C_{Surf} anodes in (a) lithium-ion storage and (b) sodium-ion storage.

Detailed descriptions of the lithium/sodium storage performances.

As shown in Fig. S17a, TiO_2/C_{Inter} exhibits the highest charge capacity of 165 mAh/g after 300 cycles at 1 C, which is about 9.7-fold of Ti-G, 1.9-fold of TiO_2-V_{Ti}/C, 7.5-fold of n-TiO_2/C and 3.0-fold of TiO_2/C_{Surf}. Similar to the lithium storage performance, TiO_2-350 also shows the highest sodium storage capacity of 135 mAh/g after 300 cycles at 1 C, which is 5.4-fold of Ti-G, 1.2-fold of TiO_2-V_{Ti}/C, 4.0-fold of n-TiO_2/C and 2.4-fold of TiO_2/C_{Surf} (Fig. S17b). Moreover, the TEM images of TiO_2/C_{Inter} after the lithium/sodium-ion cycling performance for 300 cycles (Fig. S18) show that their nanostructure is well-maintained, indicating their good structural stability.

According to the rate performance of lithium-ion storage shown in Fig. S19a, TiO_2/C_{Inter} displays high capacities of 200, 194, 185, 172, 153 and 131 mAh/g at current densities of 0.2 C, 0.5 C, 1 C, 2 C, 5 C and 10 C, respectively, and also a reversible capacity of 180 mAh/g at 0.2 C, which is obviously higher than TiO_2/C_{Surf}. For the rate performance of sodium-ion storage, TiO_2/C_{Inter} also displays high capacities of 217, 184, 168, 152, 133 and 118 mAh/g at current densities of 0.2 C, 0.5 C, 1 C, 2 C, 5 C and 10 C, and also a reversible capacity of 197 mAh/g at 0.2 C (Fig. S19b).

The excellent stability and high rate performance of TiO_2/C_{Inter} could be attributed to its unique structural features. Firstly, the very thin nanosheet could shorten the diffusion length for Li^+/Na^+, which allows for fast insertion/extraction of Li^+/Na^+.[22] Secondly, the amorphous interface between TiO_2 crystals could be a more effective structure to increase the capacity of Li^+ due to less structural confinement for the Li^+ ions in the insertion/extraction reaction.[23] Thirdly, the interlayered carbon and the titanium vacancies could endow it with good

electronic conductivity and enhance the mobility and diffusion of Li^+/Na^+.[24] These structural advantages are favorable for the increased Li^+ and Na^+ reversible capacities.

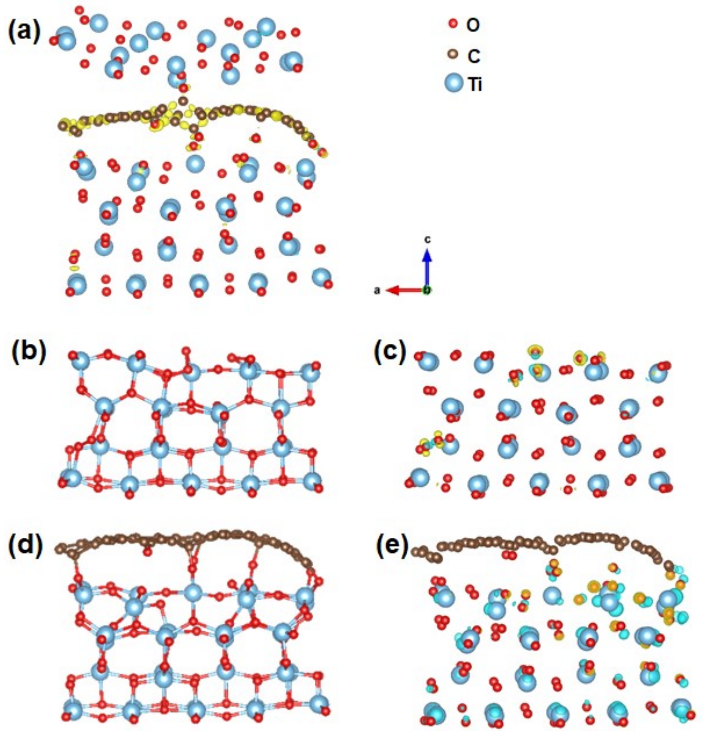

Fig. S20. The simulated model and calculated charge density difference. (a) charge density difference of the model of TiO_2 with Ti-vacancies and interlayered carbon. (b) the model of TiO_2 with Ti-vacancies and (c) corresponding charge density difference, (d) the model of TiO_2 with Ti-vacancies and surface carbon, (e) corresponding charge density difference, yellow and cyan regions in (a), (b) and (d) represent electron accumulation and depletion, respectively.

Detailed descriptions of theoretical calculations

To investigate the role of interlayered carbon in charge transfer, three models are built including TiO_2 with Ti-vacancies (TiO_2-V_{Ti}) (Fig. S20b), TiO_2 with Ti-vacancies and surface carbon (TiO_2-V_{Ti}-C_{Surf}) (Fig. S20d), and TiO_2 with Ti-vacancies and interlayered carbon (TiO_2-V_{Ti}-C_{Inter}) (Fig. 3e). Notably, an obvious charge accumulation can be observed in the interlayered carbon layer of TiO_2-V_{Ti}-C_{Inter} (Fig. S20a). Moreover, a relatively weak charge accumulation is also observed at the neighbouring oxygen atoms of both interlayered carbon and titanium vacancies, which indicates a cascade transfer path from titanium vacancies to the

interlayered carbon. The charge density difference of TiO_2-V_{Ti} show that titanium vacancies could also affect the charge distribution of the neighbouring atoms (Fig. S20c), but the influence is relatively weaker compared with that of TiO_2-V_{Ti}-C_{Inter}, which indicates that a junction of interlayered carbon and titanium vacancies is more beneficial for the charge transfer. There is no charge accumulation in the surface carbon layer in TiO_2-V_{Ti}-C_{Surf}, which indicates that interlayered carbon is more efficient for charge transfer compared with surface carbon (Fig. S20e). To have a better understanding of the charge transfer path, we further investigated the charge density difference of the section model of (010) facet in TiO_2-V_{Ti}-C_{Inter}. From Fig. 3f and 3g, it can be clearly observed that there is charge accumulation of the atoms around interlayered carbon and titanium vacancies. Therefore, they can act as bridge for the efficient charge transfer from TiO_2 lattice to titanium vacancies and to interlayered carbon.

The stability of TiO_2 with surface carbon and interlayered carbon are investigated by calculating the formation energy of different models. The calculation is based on the formula listed in Table S5. TiO_2-V_{Ti}-C_{Inter} show a lower formation energy (-45 eV) compared with TiO_2-V_{Ti}-C_{Surf} (-36.8 eV) (Table S5), which indicates that the interlayered carbon structure is relatively stable than TiO_2-V_{Ti}-C_{Surf}. Besides, the formation energy of titanium vacancies are further calculated to understand the stability of titanium vacancies in different models. To calculate the formation energy of titanium vacancies, three modes without defects are firstly constructed and opitimized. The calculation details are listed in Table S6. It can be seen that the formation energy of titanium vacancies in TiO_2-V_{Ti}-C_{Inter} model is the lowest among the three models (Table S6), which indicates that titanium vacancies are more easily to form and more stable in TiO_2-V_{Ti}-C_{Inter} structure.[25] The low formation energy of titanium vacancies in TiO_2-V_{Ti}-C_{Inter} could be attributed to the existence of interlayered carbon, which could affect the electron distribution and orbital hybridization of the neighbouring atoms. These theoritical

calcultion results demonstrate that TiO_2 with interlayered carbon and titanium vacancies has good stability, which is in correspondance with the experimental results.

Detailed description of the formation of interfacial defects expressed by Kröger-Vink notation.

The formation process can be expressed as:

$$Ti(C_4H_9O)_4 + C_3H_8O_3 \rightarrow Ti(C_3H_6O_3)_2 + 4C_4H_9OH \quad (1)$$

$$Ti(C_3H_6O_3)_2 + xO_2 \rightarrow TiO_2 + yCO_2 + 6H_2O + (6 - y)C + (2x - 2 - 2y)O_M^x \quad (2)$$

Where O_O represents lattice oxygen, $e^{'}$ represents electron, and O_M^x represents migrating oxygen of dynamic state/balance.

The formation of titanium vacancy can be described as:

$$2O_M^x \rightarrow V_{Ti}^{''''} + 2O_O + 4h^{\cdot} \quad (3)$$

Where $V_{Ti}^{''''}$ represents titanium vacancy, and $h^{\cdot}$ represents hole.

Titanium vacancy is acceptor-type defect, which means O^{2-} near titanium vacancy will change to O^- to balance the charge.

The related formation of O^- can be described as:

$$O_o + h^{\cdot} \rightarrow O_O^{\cdot} \quad (4)$$

Where $O_O^{\cdot}$ represents O^-.

The lattice charge neutrality at the interface can be described as:

$$nil \rightarrow e^{'} + h^{\cdot} \quad (5)$$

Reference

(1) Li, F.; Liu, W.; Lai, Y.; Qin, F.; Zou, L.; Zhang, K.; Li, J. *J. Alloys Comp.* **2017**, *695*, 1743.

(2) Liu, H.; Cao, K.; Xu, X.; Jiao, L.; Wang, Y.; Yuan, H. *ACS Appl. Mater. Interfaces* **2015**, *7*, 11239.

(3) Niu, P.; Wu, T.; Wen, L.; Tan, J.; Yang, Y.; Zheng, S.; Liang, Y.; Li, F.; Irvine, J. T.; Liu, G. *Adv. Mater.* **2018**, *30*, 1705999.

(4) Mondal, A.; Maiti, S.; Singha, K.; Mahanty, S.; Panda, A. B. *J. Mater. Chem.A* **2017**, *5*, 23853.

(5) Mo, R.; Lei, Z.; Sun, K.; Rooney, D. *Adv. Mater.* **2014**, *26*, 2084.

(6) Que, L. F.; Yu, F. D.; Wang, Z. B.; Gu, D. M. *Small* **2018**, *14*, 1704508.

(7) Chu, S.; Zhong, Y.; Cai, R.; Zhang, Z.; Wei, S.; Shao, Z. *Small* **2016**, *12*, 6724.

(8) Cheng, J.; Wang, B.; Xin, H. L.; Kim, C.; Nie, F.; Li, X.; Yang, G.; Huang, H. *J. Mater. Chem. A* **2014**, *2*, 2701.

(9) Zhu, Y. E.; Yang, L.; Sheng, J.; Chen, Y.; Gu, H.; Wei, J.; Zhou, Z. *Adv. Energy Mater.* **2017**, *7*, 1701222.

(10) Xu, H.; Wang, W.; Yu, G.; Qin, L.; Jiang, Y.; Ren, L.; Chen, J. *ACS Appl. Energy Mater.* **2020**, *3*, 4738.

(11) He, H.; Wang, H.; Sun, D.; Shao, M.; Huang, X.; Tang, Y. *Electro. Acta* **2017**, *236*, 43.

(12) Zhao, Y.; Xu, S.-D.; Zhang, D.; Liu, S.; Chen, L.; Zhao, H.; Wei, G. *Int. J. Electrochem. Sci.* **2017**, *12*, 1929.

(13) Van der Walt, C.; Terblans, J. J.; Swart, H. C. *Small*, **2017**, *13*, 1701829.

(14) Stevanović, V.; Lany, S.; Zhang, X.; Zunger, A. *Phys. Rev. B* **2012**, *85*, 115104.

(15) Deml, A. M.; Stevanović, V.; Holder, A. M.; Sanders, M.; O'Hayre, R.; Musgrave, C. B. *Chem. Mater.*, **2014**, *26*, 6595.

(16) Li, G.; Lian, Z.; Li, X.; Xu, Y.; Wang, W.; Zhang, D.; Tian, F.; Li, H. *J. Mater. Chem. A* **2015**, *3*, 3748.

(17) Xia, T.; Zhang, W.; Murowchick, J. B.; Liu, G.; Chen, X. *Adv. Energy Mater.* **2013**, *3*, 1516.

(18) Lazarus, M.; Sham, T. *Chem. Phys. Lett.* **1982**, *92*, 670.

(19) McCafferty, E.; Wightman, J. P. *Surf. Interface Anal.* **1998**, *26*, 549.

(20) Wang, D.; Yu, R.; Kumada, N.; Kinomura, N. *Chem. Mater.* **1999**, *11*, 2008.

(21) Chen, Y.; Tian, G.; Ren, Z.; Tian, C.; Pan, K.; Zhou, W.; Fu, H. *Eur. J. Inorg. Chem.* **2011**, *5*, 754.

(22) Chen, J. S.; Tan, Y. L.; Li, C. M.; Cheah, Y. L.; Luan, D.; Madhavi, S.; Boey, F.; Archer L.; Lou, X. W.. *J. Am. Chem. Soc.*, **2010**, *132*, 6124.

(23) Borghols, W. J., Luetzenkirchen-Hecht, D., Haake, U., Chan, W., Lafont, U., Kelder, E. M., Wagemaker, M. *J. Electro. Soc.*, **2010**, *157*, A582.

(24) Li, B., Xi, B., Feng, Z., Lin, Y., Liu, J., Feng, J., Xiong, S. *Adv. Mater.*, **2018**, *30*, 1705788.

(25) Na-Phattalung, S.; Smith, M. F.; Kim, K.; Du, M. H.; Wei, S. H.; Zhang, S. B.; Limpijumnong, S. *Phys. Rev. B* **2006**, *3*, 125205.
